# GLIS Family Zinc Finger 1 was First Linked With Preaxial Polydactyly I in Humans by Stepwise Genetic Analysis

**DOI:** 10.3389/fcell.2021.781388

**Published:** 2022-01-11

**Authors:** Jie-Yuan Jin, Pan-Feng Wu, Fang-Mei Luo, Bing-Bing Guo, Lei Zeng, Liang-Liang Fan, Ju-Yu Tang, Rong Xiang

**Affiliations:** ^1^ School of Life Sciences, Central South University, Changsha, China; ^2^ Department of Orthopaedics, Xiangya Hospital of Central South University, Changsha, China; ^3^ Hunan Key Laboratory of Animal Models for Human Diseases, School of Life Sciences, Central South University, Changsha, China; ^4^ Hunan Key Laboratory of Medical Genetics, School of Life Sciences, Central South University, Changsha, China

**Keywords:** Glis1, PPD I, SFPR2, stepwise genetic analysis, nuclear localization signal, preaxial polydactyly

## Abstract

**Background:** Preaxial polydactyly (PPD) is one of the most common developmental malformations, with a prevalence of 0.8–1.4% in Asians. PPD is divided into four types, PPD I–IV, and PPD I is the most frequent type. Only six loci (*GLI1*, *GLI3*, *STKLD1*, ZRS, pre-ZRS, and a deletion located 240 kb from *SHH*) have been identified in non-syndromic PPD cases. However, pathogenesis of most PPD patients has never been investigated. This study aimed to understand the genetic mechanisms involved in the etiology of PPD I in a family with multiple affected members.

**Methods:** We recruited a PPD I family (PPD001) and used stepwise genetic analysis to determine the genetic etiology. In addition, for functional validation of the identified *GLIS1* variant, *in vitro* studies were conducted. *GLIS1* variants were further screened in additional 155 PPD cases.

**Results:** We identified a *GLIS1* variant (NM_147193: c.1061G > A, p.R354H) in the PPD001 family. *In vitro* studies showed that this variant decreased the nuclear translocation of GLIS1 and resulted in increased cell viability and migration. RNA sequencing revealed abnormal *TBX4* and *SFRP2* expression in 293T cells transfected with mutant GLIS1. Additionally, we identified a *GLIS1* variant (c.664G > A, p.D222N) in another PPD case.

**Conclusion:** We identified two *GLIS1* variants in PPD I patients and first linked *GLIS1* with PPD I. Our findings contributed to future molecular and clinical diagnosis of PPD and deepened our knowledge of this disease.

## Introduction

Preaxial polydactyly (PPD) is one of the most common developmental malformations, occurring in 0.8–1.4% of Asians (Evanson et al., 2016). PPD is phenotypically divided into four types: PPD I–IV, and PPD I (OMIM_174400) is the most common type in many populations (Handforth, 1950). PPD I is characterized by the duplication of one or more of the skeletal components of biphalangeal thumbs. The severity varies from a mere broadening of the distal phalanx with a slight bifurcation at the tip to a full duplication of the thumb, including the metacarpals (Lange and Muller, 2017). Currently, six loci have been identified in nonsyndromic PPD cases, including *GLI1*, *GLI3*, *STKLD1*, the zone of polarizing activity (ZPA) regulatory region (ZRS), which is a limb-specific enhancer of *SHH* that is located within intron five of *LMBR1*, the pre-ZRS region (a noncoding evolutionary conserved sequence 500 bp upstream of the ZRS), and a deletion located 240 kb from the *SHH* promotor. Genetic etiologies in most PPD patients have never been evaluated (Petit et al., 2016; Potuijt et al., 2018; Ullah et al., 2019; Umair et al., 2019; Xu et al., 2020; Baas et al., 2021).

*GLIS1* encodes GLIS family zinc finger protein 1, a transcriptional activator and repressor, though it primarily functions as a transcriptional activator. GLISs, together with GLIs and ZICs, constitute the Krüppel-like zinc finger family, which is characterized by classical Cys2-His2 zinc fingers (Lee et al., 2017). *In situ* hybridization of mouse embryos showed that Glis1 is expressed primarily in tissues with mesodermal lineages, including limb buds. Glis1 was first observed in the anterior-proximal mesenchyme during early limb bud development in mice (E9.5) and then extends along the entire apical ectodermal ridge (AER) at E11.5 (Kim et al., 2002). The expression pattern is similar to Gli3 expression, suggesting that Glis1 may play an important role in mouse limb patterning. However, Glis1 function in limb development remains ambiguous. In addition, GLIS1 is commonly used to generate human induced pluripotent stem cells (iPSCs), where GLIS1 can replace c-MYC and interact with OCT3/4, SOX2, and KLF4 to markedly enhance iPSC generation and promote multiple pro-reprogramming pathways, including NANOG, WNT, and the mesenchymal–epithelial transition (Maekawa et al., 2011). In breast cancers, GLIS1 and CUX1 cooperate to stimulate the WNT signaling pathway (Vadnais et al., 2014). *GLIS1* confers susceptibility to mitral valve prolapse (MVP). The single nucleotide polymorphism (SNP), rs1879734, which is located in the first intron of *GLIS1*, is specifically associated with MVP (Yu et al., 2019).

Thus far, no study has reported a correlation between *GLIS1* and PPD in humans. In this study, we identified a heterozygous missense variant in *GLIS1* (NM_147,193: c.1061G > A, p.R354H) responsible for PPD I in a large Chinese family by stepwise genetic analysis and functional verification. This correlation was verified in other PPD cases. In summary, our study was the first to link *GLIS1* variants with PPD and contributed to genetic screening for PPD patients, thereby deepening our knowledge of PPD.

## Materials and Methods

### Patients and Subjects

**FIGURE 1:**
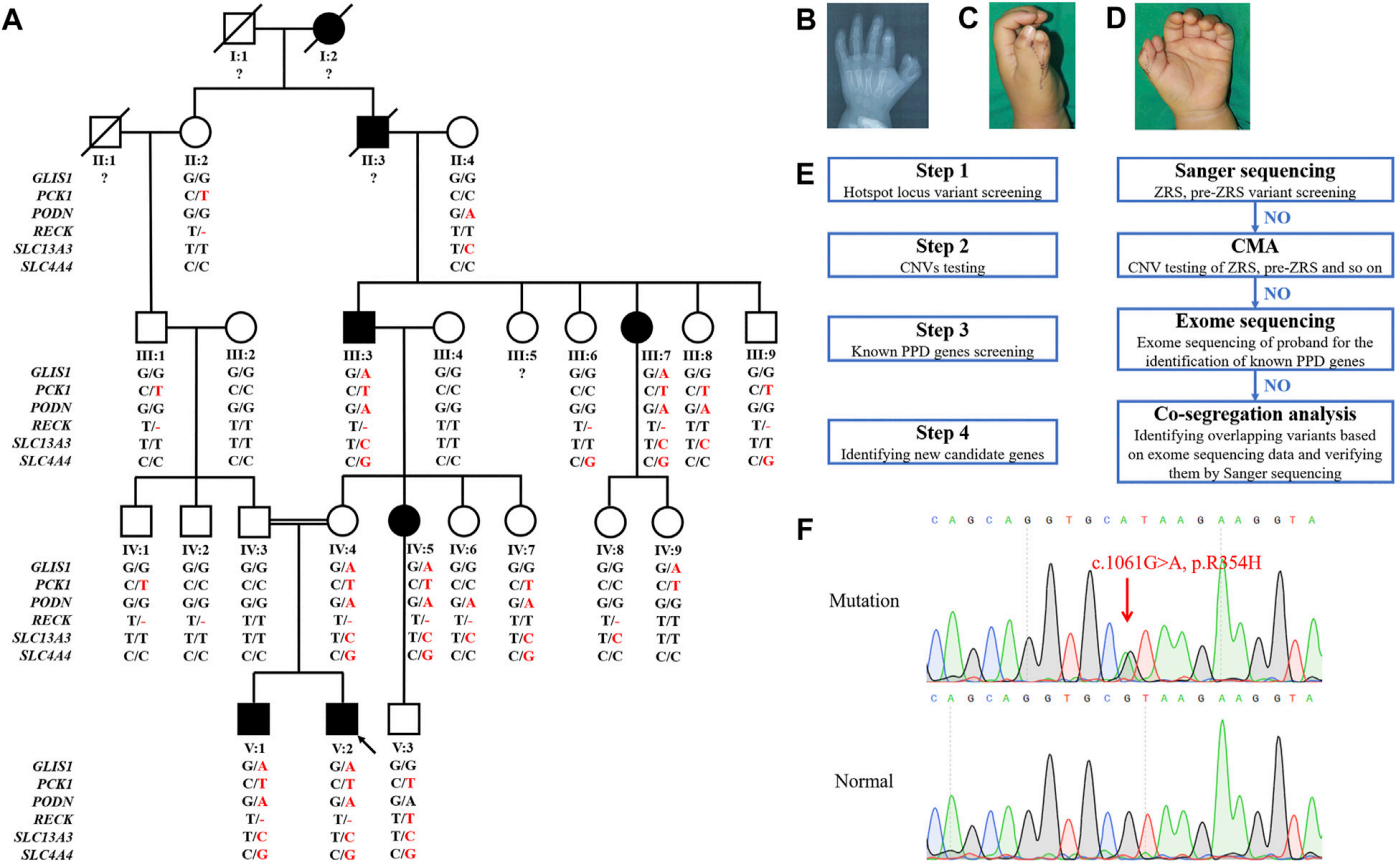
Pedigree, symptoms, and stepwise genetic analysis of the PPD001 family. **(A)** Pedigree of PPD001 family with seven patients. The black symbols represent the affected members, and the arrow indicates the proband. Genotypes are identified by letters and a slash, with red letters representing variants. Question marks represent unknown data or symptoms. **(B–D)** The proband was affected by PPD I. **(E)** The stepwise genetic analysis strategy. **(F)** Sanger sequencing results of the *GLIS1* variant (c.1061G > A, p.R354H).

This research was approved by the Review Board of Xiangya Hospital of Central South University. We recruited a five-generation Chinese family (PPD001) with PPD I (Figure 1A). Additional 155 PPD families or sporadic cases were recruited to screen the candidate gene. Written informed consent was obtained from the patients and their guardians. Blood was collected from the probands and blood relations. Genomic DNA (gDNA) was extracted using DNeasy Blood & Tissue Kit (Qiagen).

### Polymerase Chain Reaction and Sanger Sequencing

The sequences of ZRS regulatory region and pre-ZRS region were obtained from NCBI (https://www.ncbi.nlm.nih.gov/gene/105804841). Primers were designed by IDT (http://sg.idtdna.com/Primerquest/Home/Index) (Supplementary Table S1). Target sequences were amplified using polymerase chain reaction (PCR) and detected by Biosune Company Limited (Shanghai China).

### Chromosomal Microarray Analysis

Chromosomal microarray analysis (CMA) was conducted in two affected individuals (V:1 and V:2 of the PPD001 family) as described by Jin et al. (2019), with slight modifications where necessary (Jin et al., 2019).

### Exome Sequencing and Co-Segregated Analysis

Berry Genomics Company Limited (Chengdu, China) performed exome capture, high-throughput sequencing, and common filtering, as described previously (Jin et al., 2020). After filtering common variants (allele frequency >0.05) from the 1000G (https://www.genome.gov/27528684/1000-genomes-project/) and GnomAD (http://gnomad.broadinstitule.org), unique SNPs and insertions/deletions (indels) were identified. MutationTaster (http://www.mutationtaster.org/), PolyPhen-2 (http://genetics.bwh.harvard.edu/pph2/), and SIFT (http://provean.jcvi.org/index.php) were used to predict variant pathogenicity. Gene function, inheritance pattern, and clinical phenotype were annotated according to OMIM (https://www.omim.org). Pathogenicity was classified according to the American College of Medical Genetics classification (Richards et al., 2015).

Eight subjects of the PPD001 family (III:3–4, III:7, IV:3-5, and V:1–2) were subjected to exome sequencing. First, we screened unique SNPs that were predicted to be disease-causing. Then, we selected variants in patients (III:3, III:7, IV:5, V:1, and V:2) and IV:4, which were absent in III:4 and IV:3. We determined whether these overlapping variants were PPD causative or candidate genes (classical limb bud pattern-related genes). Finally, we selected variants that may be associated with PPD and conducted PCR and Sanger sequencing in all family members for co-segregation analysis.

### Mutagenesis of GLIS1-1061G > A

pcDNA3.1-flag-GLIS1 plasmids were obtained from WZ Biosciences Inc. (Jinan, China). Mutagenesis of GLIS1-1061G > A (pcDNA3.1-flag-mGLIS1) was performed using Fast Mutagenesis Kit V2 (Vazyme). GLIS1 mutant and wild-type plasmids were validated using Sanger sequencing.

### Cell Culture, Transfection, Transwell, and CCK8 Test

293T cells were obtained from the National Collection of Authenticated Cell Cultures. Cells were seeded in 6-well plates (5 × 10^5^ cells per well) and transfected with pcDNA3.1-flag-GLIS1 vectors or pcDNA3.1-flag-mGLIS1 vectors (2 μg per well) using Lipofectamine 3,000 (Invitrogen) for 24 h, or seeded in 24-well plates (1 × 10^5^ cells per well) with 0.4 μg vectors per well. Transwell assays using 3,000 cells were performed 24 h after transfection using transwell chambers (Costar). Five thousand transfected cells were seeded into a 96-well plate and cultured for 24 h. Then, CCK8 assays were performed using CCK-8 Cell Proliferation and Cytotoxicity Assay Kit (Solarbio).

### Protein Extraction and Western Blot

Nuclear and cytosolic proteins from 293T cells were extracted using Nuclear Protein Extraction Kit (Solarbio). SDS-PAGE was performed using a 12.5% PAGE Gel Rapid Preparation Kit (Yeasen). Lysates were mixed with SDS-gel sample buffer and heated at 90°C for 10 min. Then, the protein samples were loaded onto the PAGE gels. After electrophoresis, the bands were electrophoretically transferred onto a nitrocellulose membrane. After blocking with 1% bovine serum albumin (BSA) in Tris-buffered saline, the membranes were incubated with anti-FLAG primary antibody (Proteintech, 20543-1-AP, 1:5,000), anti-GAPDH primary antibody (Proteintech, 10494-1-AP, 1:5,000), and anti-Histone-H3 polyclonal antibody (Proteintech, 17168-1-AP, 1:1,000). Chemiluminescent signals were scanned, and integrated density values were calculated with a chemiluminescent imaging system (Alpha Innotech).

### Immunofluorescence

Immunofluorescence was performed on cells 48 h after transfection. Cells were fixed with 4% paraformaldehyde for 15 min and blocked in 5% BSA in PBS for 30 min at room temperature. The samples were permeabilized with Triton X-100 (0.15%) and incubated overnight at 4°C with anti-FLAG primary antibody (Proteintech, 20543-1-AP, 1:1,000) in PBS containing 5% BSA. The cells were then incubated with mouse Alexa 488-conjugated secondary antibody (Invitrogen, A-11094, 1:400) for 1 h at room temperature. Nuclei were stained with DAPI (Solarbio), and the cells were examined using Leica SP5 confocal microscope (Leica).

### Total RNA Extraction, Transcriptome Sequencing, and Real-Time PCR

Total RNA was extracted from cells 24 h after transfection using RNA Extraction Kit (Qiagen) and stored at −80°C. Transcriptome sequencing was performed by Berry Genomics Company Limited using Illumina HiSeq 4,000 sequencing platform (Illumina). Genes with *p* < 0.05 and -1 < log2 fold change >1 were considered differentially expressed genes.

Total RNA was reverse transcribed using RevertAid First Strand cDNA Synthesis Kit (Thermo) and then subjected to real-time PCR by 2xSYBR Green qPCR Mix (Thermo).

### Mutant Modeling

The GLIS1 protein structure (Q8NBF1) was downloaded from SWISS-MODEL database (https://swissmodel.expasy.org/). PyMol was used to build mutant models according to the wide-type structure.

### Statistical Analysis

Statistical analyses were conducted using SPSS (version 18.0; IBM, Inc.). The data were first tested for normal distribution using the Kolmogorov-Smirnov test. Differences between groups were identified using Student’s *t*-test or analysis of variance (ANOVA) if the data were normally distributed, and the Mann-Whitney *U* test or Kruskal-Wallis test was used for non-normally distributed data. Categorical data were compared using the chi-squared test.

## Results

### Clinical Features of the Five-Generation PPD I Family

We identified a five-generation Chinese family with PPD I (PPD001, Figure 1A). The proband (V:2) was a 3-year-old boy, who was diagnosed with PPD I (Figures 1B–D). Tracing his family history, we found seven PPD I patients (including two deceased individuals) across five generations. All affected members (III:3, III:7, IV:5, V:1, and V:2) had PPD I (Table 1). No conspicuous phenotypes were observed in the fingers or toes of other subjects.

**TABLE 1:** Phenotypes of parts family members in PPD001 family.

Family member	I:2	II:3	III:3	III:7	IV:5	V:1	V:2	IV:4	IV:9
Gender	F	M	M	F	F	M	M	F	F
Age (years)	Unknown	73	57	51	28	8	3	32	23
Preaxial polydactyly	+	Left hand	Left hand	Left hand	Left hand	Left hand	Left hand	-	-
Triphalangeal thumb	Unknown	Unknown	-	-	-	-	-	-	-
Other phenotypes of upper lambs	Unknown	-	-	-	-	-	-	-	-
Lower limbs	-	-	-	-	-	-	-	-	-
Other	Unknown	Unknown	Hypertension	Diabetes	-	-	-	-	Arrhythmia

F, female; M, male; +, observed phenotype; -, no symptoms.

### Genetic Analysis of the PPD001 Family

Stepwise strategies were used to identify the genetic etiology of the PPD001 family (Figure 1E). We screened variants in ZRS and pre-ZRS regions by Sanger sequencing, but did not detect variants in the proband. CMA results of the proband and his brother (V:1) showed an unfavorable copy number variation (CNV; Supplementary Table S2). Then, we conducted exome sequencing to detect known PPD-related genes in the proband, but did not identify variants in these genes, indicating that there was a novel gene variant causing PPD in this family.

To further identify pathogenic variants, we selected five affected (III:3, III:7, IV:5, V:1, and V:2) and three normal family members (III:4, IV:3, and IV:4) for exome sequencing. Based on the family pedigree, we hypothesized that PPD in this family was transmitted via autosomal dominant inheritance and that IV:4 was a carrier. Thus, we screened overlapping variants in several patients and IV:4. Finally, variants in six genes were identified (Table 2). After co-segregation of all subjects, a *GLIS1* variant (NM_147193: c.1061G > A, p.R354H) was retained (Figures 1A,F). Thus, we reasoned that the *GLIS1* variant could be the major etiological factor in this family.

**TABLE 2:** Overlapping variants identified in PPD001 family by WES.

Gene	Variant	Mutation taster	PolyPhen-2	SIFT	MUpro	GnomAD	OMIM clinical phenotype	American College of medical genetics classification
*GLIS1*	NM_147193: c.1061G > A, p.R354H	D	D/B	D	Decrease stability	0.00009	-	Likely pathogenic (PS3, PP1, PP3, PP5)
*PCK1*	NM_002591: c. 1576C > T, p.L526F	D	D/D	D	Decrease stability	0.00005	AR, Phosphoenolpyruvate carboxykinase deficiency, cytosolic	Uncertain significance (PP3, PP5, BS4)
*PODN*	NM_001199080: c.781G > A, p.V261M	D	D/D	T	Decrease stability	0.00001	-	Uncertain significance **(**PP3, PP5, BS4)
*RECK*	NM_021111: c.789delT, p.C263fs	D	−/−	-	-	-	-	Uncertain significance (PM2, PP3, PP5, BS4)
*SLC13A3*	NM_022829: c.857T > C, p.M286T	D	D/D	D	Decrease stability	0.00011	AR, Leukoencephalopathy, acute reversible, with increased urinary alpha-ketoglutarate	Uncertain significance (PP3, PP5, BS4)
*SLC4A4*	NM_003759: c.1435C > G, p.L479V	D	D/D	D	Mildly decrease stability	-	AR, Renal tubular acidosis, proximal, with ocular abnormalities	Uncertain significance (PM2, PP3, PP5)

D, disease causing; T, tolerated; B, benign; AR, autosomal recessive.

PolyPhen-2, results showed the prediction of HumDiv and HumVar in turn.

### Functional Analysis of the GLIS1 p.R354H Variant

**FIGURE 2:**
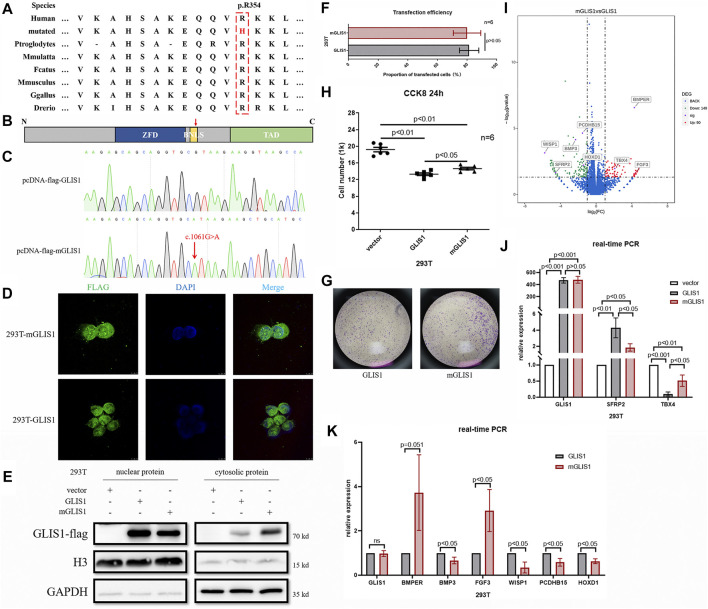
Functional analysis of variant p.R354H *in vitro*. **(A)** Species conservation analysis of the mutant amino acid of GLIS1. **(B)** Schematic diagram of GLIS1 structure. Red arrow indicates variant p.R354H. **(C)** Sequencing results of GLIS1-1061G > A mutagenesis plasmids. **(D)** Immunofluorescence results demonstrating that mutant GLIS1 had increased cytoplasmic retention and reduced the proportion of nuclear protein. **(E)** Western blot showed that compared with wild-type, nuclear protein of the mutant GLIS1 was reduced, and cytosolic protein was increased. **(F)** The transfection efficiency of GLIS1/mGLIS1 plasmids in 293T cells. **(G, H)** CCK8 and transwell results showing that mutant GLIS1 promoted cell viability and migration. “*p* < 0.05” presents data with statistical significance, “*p* > 0.05” presents data without statistical significance, and “n” represents the number of cell samples. **(I)** A volcano plot based on RNA sequencing data. **(J, K)** Validation of RNA sequencing data by the real-time PCR. “*p* < 0.05” presents data with statistical significance, and “*p* > 0.05” presents data without statistical significance.

Amino acid sequence alignment analysis showed that p.R354 in GLIS1 was highly conserved throughout evolution (Figure 2A). We used UniProtKB database (https://www.uniprot.org/uniprot/Q8NBF1) to predict that the variant p.R354H occurred in a bilateral nuclear localization signal (BNLS, Figure 2B). We hypothesized that the mutant BNLS hampered GLIS1 transportation to the nucleus and damaged GLIS1 function as a transcription factor. Wild-type GLIS1 plasmids (pcDNA-flag-GLIS1) were mutated into GLIS1-1061G > A plasmids (pcDNA-flag-mGLIS1, Figure 2C). Both wild-type and mutant plasmids were transfected into 293T cells. Subsequent immunofluorescence and Western blot analysis demonstrated that compared with wild-type, the mutant GLIS1 had increased cytoplasmic retention, which reduced its proportion in the nucleus (Figures 2D,E). CCK8 and transwell assays showed that mutant GLIS1 promoted cell viability and migration (Figures 2F,G). Transcriptome sequencing data showed abnormal *TBX4* and *SFRP2* expression in 293T cells expressing mutant GLIS1, and these results were verified by real-time PCR (Figures 2I,J). TBX4 and SFRP2 play important roles in limb development(Ikegawa et al., 2008; Jain et al., 2018). Furthermore, p.R354H also led to alterations in the expression of other 237 genes, including *BMP3*, *BMPER*, *PCDHB15*, *WISP1*, *HOXD1*, and *FGF3* (Figures 2I,K). Thus, we confirmed that the *GLIS1* variant decreased nuclear translocation, resulting in an increase in cell viability and migration.

### Screening GLIS1 Variants in Sporadic or Familial Cases

**FIGURE 3:**
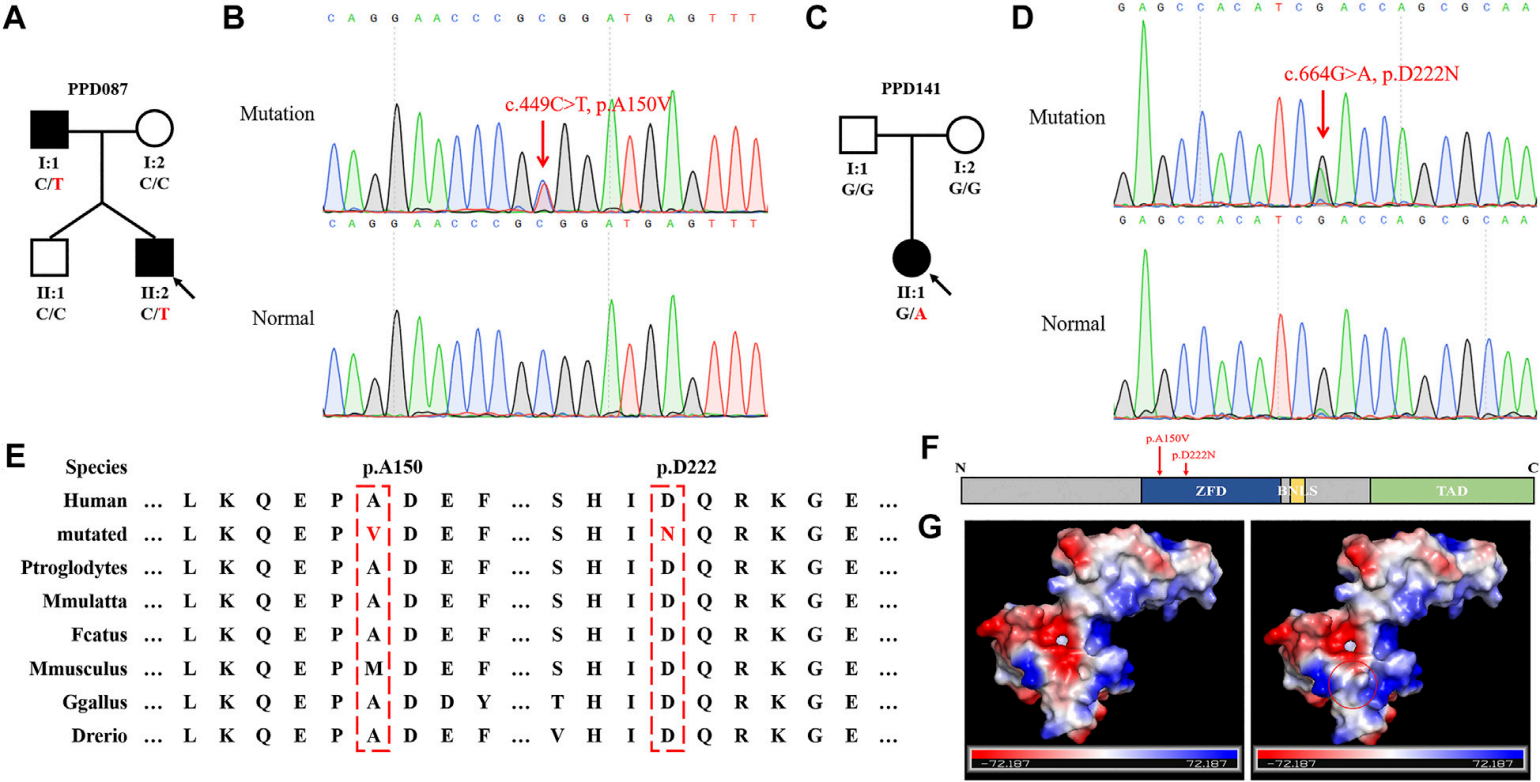
*GLIS1* variants identified in PPD I cases. **(A, C)** Pedigrees of PPD I families. The black symbols represent affected members, and the arrow indicates the proband. Genotypes are identified by letters and a slash, with red representing variants. **(B, D)** Sanger sequencing results of *GLIS1* variants. **(E)** Species conservation analysis of mutant amino acid sites of GLIS1. **(F)** Schematic diagram of GLIS1 structure. The red arrows indicate variants. **(G)** Protein models of GLIS1 with or without the variant (p.D222N). The red circle indicates the variant site.

We recruited additional 155 sporadic or familial cases with PPD to further verify the correlation between *GLIS1* variants and PPD. Two *GLIS1* variants (c.449C > T, p.A150V and c.664G > A, p.D222N) were identified in two PPD families (PPD087 and PPD141; Figures 3A–D; Table 3). The variant (c.449C > T, p.A150V) was identified in two patients of the PPD087 family, with a minor allele frequency (MAF) of 0.00043. p.A150 was highly conserved and located in zinc finger domains (ZFDs), but bioinformatics analysis predicted that p.A150V was benign (Figures 3E,F; Table 3). Thus, it was considered a polymorphism. The variant (c.664G > A, p.D222N) arose *de novo* and was detected in a baby with PPD I that was conceived *in vitro*. The variant was predicted to be disease-causing (Table 3). p.D222 was located in ZFDs with high evolutionary conservation (Figures 3E,F). According to three-dimensional modeling of GLIS1 protein, p.D222N caused surface charge modifications that may impact the ability to bind target DNA sequences (Figure 3G). We reasoned that the *GLIS1* variant (c.664G > A, p.D222N) may be the genetic etiology in the PPD141 family, and *GLIS1* may be a novel causative gene of PPD I.

**TABLE 3:** Phenotypes and genotypes of PPD patients with *GLIS1* (NM_147193) variants.

Patient	Phenotype	*GLIS1* variant	MutationTaster	PolyPhen-2	SIFT	MUpro	GnomAD	American college of medical genetics classification
PPD087-I:1	PPD I on the right hand	c.449C > T, p.A150V	P	B/B	T	Decrease stability	0.00043	Uncertain significance (PP1, BP4)
PPD087-II:2	Bilateral PPD I							
PPD141-II:1	PPD I on the right hand	c.664G > A, p.D222N	D	D/B	D	Decrease stability	0.00000	Likely pathogenic (PS2, PM2, PP3)

PPD I, preaxial polydactyly type I; D, disease causing; T, tolerated; P, polymorphism; B, benign.

PolyPhen-2, results showed the prediction of HumDiv and HumVar in turn.

## Discussion

PPD I is a frequent developmental defect worldwide, while disease-causing variants have only been identified in a few patients (Ullah et al., 2019; Umair et al., 2019; Xu et al., 2020). Genetic etiologies of most cases remain to be studied. In this study, we aimed to understand the genetic mechanisms involved in the etiology of PPD I in the PPD001 family through stepwise genetic analysis, a classic variant screening strategy. Six variants in six genes were detected, including *GLIS1*, *PCK1*, *PODN*, *RECK*, *SLC13A3*, and *SLC4A4*. The *PODN* and *SLC13A3* variants were derived from II:4. II:2 and her normal descendants (III:1, IV:1, and IV:2) harbored the *PCK1* and *RECK* variants, but these individuals did not have limb malformations. Thus, we excluded these four candidate genes from the analysis. Moreover, five members (III:6, III:9, IV:4, IV:7, and V:3) presented with a heterozygous variant of *SLC4A4*, which is responsible for isolated proximal renal tubular acidosis, an autosomal recessive disease (Igarashi et al., 1999). In contrast, only two normal members carried the *GLIS1* variant (c.1061G > A, p.R354H). Given that PPD is associated with environmental effects, gender differences, and genetic factors, we speculated that the *GLIS1* variant carriers without PPD (IV:4 and IV:9) may be due to individual differences, the lower possibility of PPD in females, and the variant being the main cause of PPD rather than the only cause. Hence, we determined that the *GLIS1* variant was more likely to cause PPD in this family. Subsequent genetic screening in 155 cases further verified that *GLIS1* was associated with PPD.

GLIS1 contains a DNA-binding domain (DBD) consisting of five Cys2-His2 ZFDs, a BNLS, and a transcription activation domain (TAD) (Jetten, 2018). Variant p.R354H was located in the BNLS and affected GLIS1 translocation to nucleus, causing cytoplasmic retention. We speculated that p.R354H may decrease GLIS1 nuclear translocation efficiency and may impede GLIS1 function as a transcription factor. Variant p.D222N occurred in ZFDs and caused the substitution of an acidic amino acid with a basic residue. Based on the three-dimensional modeling results, the variant would change surface charge, which may affect the capacity of DNA binding.

GLIS1 is a regulator of mesenchymal multipotency *in vitro* and plays a crucial role in cell reprogramming (Scoville et al., 2017; Gerard et al., 2019). Several studies showed that GLIS1 acted on multipotent stem cells. In mouse embryos, GLIS1 is expressed primarily in the mesodermal lineages, including craniofacial regions, branchial arches, myotomes, and limb buds (Kim et al., 2002). We hypothesized that GLIS1 participated in the development of mesoblast-derived embryonic structures. PPD occurrence is accompanied by an increase in cell proliferation and migration in the anterior-proximal region of limb buds (Crick et al., 2003). Variants in *GLI3* and ZRS cause PPD by inducing the anterior ectopic expression of SHH (Deng et al., 2015; Xu et al., 2020). SHH promotes cell proliferation and migration *in vivo* and *in vitro* (Hayes et al., 2001; Wang et al., 2019). We confirmed increased 293T cell viability and migration after transfection with the variant p.R354H. However, we did not observe SHH overexpression. Additionally, there is no report on the correlation between GLIS1 and SHH. We speculated that GLIS1 may be downstream of SHH, analogous to GLI1 and SFRP2; however, this hypothesis should be further validated (Ikegawa et al., 2008; Jeon and Seong, 2016). Sfrp2 inactivation can inhibit apoptosis in the central interdigital spaces of mice (Ikegawa et al., 2008). Decreased SFRP2 expression induced by mutant GLIS1 may better protect cells from apoptosis and may be responsible for increased cell viability in mGLIS1 293T cells.

GLIS1, GLIS2, and GLIS3 constitute a subfamily of Krüppel-like zinc finger proteins (Kang et al., 2010). As transcription factors, GLISs interact with target genes with GLIS-binding sites (GLISBS). GLISBS is a G-rich DNA sequence, (G/C)TGGGGG(A/C). The GLI and ZIC binding sites are G-rich DNA response elements similar to GLISBS (Jetten, 2018). In fact, ZFDs are highly homologous among GLISs and GLIs (Kang et al., 2010). Therefore, GLISs, GLIs, and ZICs might compete for these binding sites. For example, GLIS2 can compete with GLI1 for the same binding site as *WNT4*, making antagonistic action against GLI1 (Vasanth et al., 2011). Moreover, these Krüppel-like zinc finger proteins might interact and form heterodimers, such as the GLI and ZIC subfamilies (Koyabu et al., 2001). We speculated that GLIS1 can interact with GLIs or act on genes activated by GLIs during limb development. GLIs are downstream proteins of SHH in SHH signaling pathway and deeply involve in finger developments. SFRP2, a known SHH/GLIs target gene, was downregulated in 293T cells with mutant GLIS1 (p.R354H), hinting at the effect of GLIS1 on the SHH/GLIs signaling pathway (Milla et al., 2012). A single *Sfrp2* deletion triggered syndactyly and preaxial synpolydactyly in mice, and a loss of Sfrp2 function resulted in brachy-syndactyly in mice through the Wnt signaling pathway (Ikegawa et al., 2008; Morello et al., 2008). The *GLIS1* variants (c.1061G > A, p.R354H; c.664G > A, p.D222N) identified in the present study may lead to PPD by downregulating SFRP2, although there is no report about GLIS1 regulating SFPR2.

In cancers and during cellular reprogramming, GLIS1 activates the WNT signaling pathway, especially several WNTs (Maekawa et al., 2011). In this study, mutant GLIS1 upregulated BMPER, TBX4, and FGF3, while downregulating PCDHB15, BMP3, WISP1, and HOXD1. Tbx4 triggers the initiation of vertebrate limb development through activation of the Wnt/Fgf signaling cascade (Takeuchi et al., 2003). PCDHB15, WISP1, and FGF3 are involved in the WNT or FGF signaling pathways (Wong et al., 2020). BMPER regulates BMP2/4, and BMP3 antagonizes BMP2 to induce osteoprogenitor differentiation and ossification (Daluiski et al., 2001; Moser et al., 2003). BMP2 is crucial for finger outgrowth, and its variants lead to finger deformities (Dathe et al., 2009). Although WNTs did not show prominent differences in our RNA sequencing data, the expression of many WNT signaling pathway-related genes was altered in 293T cells with mutant GLIS1. This may be because different cell lines have distinct expression profiles and GLIS1 target genes may be variable in different cell lines.

In this study, we detected *GLIS1* variants only in two PPD I families (PPD001 family and PPD141 family). There was no evidence of a correlation between *GLIS1* and other PPD types, though this conclusion required further study. The PPD001 family followed an incomplete autosomal dominant inheritance pattern, in which two *GLIS1* variant carriers (IV:4 and IV:9) had no apparent limb malformations. These findings suggested that genetic factors acted in concert with environmental effects. Genome-wide association studies (GWAS) had identified *GLIS1* as a susceptibility gene for MVP, but none of carriers with GLIS1 variants in this study had MVP (Yu et al., 2019).

## Conclusion

In summary, we first associated *GLIS1* with PPD I in humans by stepwise genetic analysis and examined the pathogenic potential of *GLIS1* variants (c.1061G > A, p.R354H; c.664G > A, p.D222N) *in vitro* or by three-dimensional modeling. There are only a few known causative genes or enhancers of PPD in humans, and our research further helped us understand this disease and contributed to future molecular and clinical diagnosis of PPD. Further study should be performed to determine the potential mechanism by which GLIS1 defects cause PPD, which will be beneficial for future treatments. Given that Sfrp2 deletion is associated with limb defects in mice, and that our *in vitro* experiments suggested that GLIS1 can mediate SFRP2, we hypothesized that GLIS1 upregulated SFRP2 expression. Our findings hinted that GLIS1 defects may trigger reduction in SFRP2 levels to lead to PPD.

## Data Availability

The original contributions presented in the study are included in the article/Supplementary Material. Further inquiries can be directed to the corresponding authors.
