# Supplementary material for: GLIS Family Zinc Finger 1 was First Linked With Preaxial Polydactyly I in Humans by Stepwise Genetic Analysis
Source: Front Cell Dev Biol. 2022 Jan 11;9:781388. doi: 10.3389/fcell.2021.781388 (PMC8787328; doi:10.3389/fcell.2021.781388)
Supplement: Supplementary file 1 [file Table1.docx]

**Supplementary Table 1**. Primer pairs of ZRS and pre-ZRS.

| **Primer** | **Sequences (5’→3’)** | **Primer** | **Sequences (5’→3’)** |
| --- | --- | --- | --- |
| PreZRS-ZRS 1f | GGTGGAAGCGAAGAGTTCTG | PreZRS-ZRS 1r | TGACAAAATTAATGTCATTTCCTG |
| PreZRS-ZRS 2f | TCTGGCCAGTGTTTAAATGG | PreZRS-ZRS 2r | TGGATCATCAGTGGCAAAAA |
| PreZRS-ZRS 3f | CCACTATGTTAATTCAAACAGGACA | PreZRS-ZRS 3r | TCCCAGAAAAATCTTGGGTTT |
| PreZRS-ZRS 4f | TTTGAGCCCTTTCATGTTGT | PreZRS-ZRS 4r | TCTAAATTCCAAGATAAAAGTTAGCTG |
| GLIS1 f | CCCATGTTACAGAAGCCGTA | GLIS1 r | GAACGGATGGACGAACAGT |
| PCK1 f | GAGAGAGAGAGAGAGAAAGAGAGAG | PCK1 r | CCATCGATCCGGTTGAACAT |
| PODN f | GTGTACCTGCACAACAACAAG | PODN r | GTTTCTGCCCACTTCTCCA |
| RECK f | TGCTGTGACAGAGCTGAAGA | RECK r | TGTAGAGGGAGGAGGGTGTA |
| SLC13A3 f | TTCCATGGGACCCTCTCATT | SLC13A3 r | CGTACAGGAAGGAGATCCAGAG |
| SLC4A4 f | TGCCTAGTGACATCACAAATGA | SLC4A4 r | TCTGAAACAAGTGGTGACTACC |
| GLIS1 1-1f | GGCTTGTCCATCCCTTGAT | GLIS1 1-1r | GGCTCCGGAGTCCATTTAC |
| GLIS1 1-2f | CGACGTCACCTCCATCATC | GLIS1 1-2r | GATGTGGCTCTTCTCGATGT |
| GLIS1 1-3f | GAGCTCTTTGGGCCTCAC | GLIS1 1-3r | CCACAGCATGGGTCCTTT |
| GLIS1 2f | CACCAGGCCTTTCCAGATAA | GLIS1 2r | GGCCAGGGATAACTGCTAAA |
| GLIS1 3f | GGATGGTGGTGGAATGAAGA | GLIS1 3r | GGACGAACAGTGGCATACA |
| GLIS1 4f | CTCAAAGGACCTGGACTAACAA | GLIS1 4r | GATTGGAAGTAGGGAACCAGAG |
| GLIS1 5f | TTCTCCATCATCAGGCACAC | GLIS1 5r | AAGCCAGTACGGTCTACTCA |
| GLIS1 6f | CTATTGTAAGTCGGAGCAGGTC | GLIS1 6r | GGGTCCCTAAGTGGGAATTG |
| GLIS1 7f | TCTCTCTCTCACACCCACAA | GLIS1 7r | GACCTCTCTGAGCCTCTACTT |
| GLIS1 8f | CCTGGAACTGGGAAGCAT | GLIS1 8r | TGGCACTCCTGCTAGGT |
