# Supplementary material for: GLIS Family Zinc Finger 1 was First Linked With Preaxial Polydactyly I in Humans by Stepwise Genetic Analysis
Source: Front Cell Dev Biol. 2022 Jan 11;9:781388. doi: 10.3389/fcell.2021.781388 (PMC8787328; doi:10.3389/fcell.2021.781388)
Supplement: Supplementary file 2 [file Table2.docx]

**Supplementary Table 2**. Overlapping CNVs were detected in V:1 and V:2 by CMA.

| **CNV** | **Position** | **Size** | **Value** | **Gene** |
| --- | --- | --- | --- | --- |
| 7p22.3 duplication | chr7:44935-89165 | 44.2 kb | 3 | No |
| 13q21.1 deletion | chr13:58587289-58599031 | 11.7 kb | 0 | No |
| 15q21.1 deletion | chr15:46845278-46880050 | 34.8 kb | 1 | No |

CNV, copy number variation; CMA, chromosomal microarray analysis; No, No gene in this locus.
